# Voluntary participation and comprehension of informed consent in a genetic epidemiological study of breast cancer in Nigeria

**DOI:** 10.1186/1472-6939-15-38

**Published:** 2014-05-13

**Authors:** Patricia A Marshall, Clement A Adebamowo, Adebowale A Adeyemo, Temidayo O Ogundiran, Teri Strenski, Jie Zhou, Charles N Rotimi

**Affiliations:** 1 Department of Bioethics, School of Medicine, Room TA 227Case Western Reserve University10900 Euclid Avenue Cleveland, Ohio 44106-4976 Cleveland, USA; 2 Department of Epidemiology and Public Health, Institute of Human Virology and Greenebaum Cancer Center of the University of Maryland School of Medicine, Baltimore Howard Hall Suite 200, 660 W. Redwood Street, Baltimore, MD 21201, USA; 3 Center for Research on Genomics and Global Health, National Institutes of Health, Building 12A, Room 4047, 12 South Dr, MSC 5635, Bethesda, MD, 20892-5635, USA; 4 Division of Oncology, Department of Surgery, University College Hospital, Ibadan, Nigeria; 5 Feinberg School of Medicine, Northwestern University, 750 N Lake Shore Dr, Chicago, IL 60611, USA

**Keywords:** Informed consent, Genetic epidemiological research, Breast cancer, Nigeria

## Abstract

**Background:**

Studies on informed consent to medical research conducted in low or middle-income settings have increased, including empirical investigations of consent to genetic research. We investigated voluntary participation and comprehension of informed consent among women involved in a genetic epidemiological study on breast cancer in an urban setting of Nigeria comparing women in the case and control groups.

**Methods:**

Surveys were administered in face-to-face interviews with 215 participants following their enrollment in the genetic study (106 patients, 109 controls). Audio-taped in-depth interviews were conducted with a sub-sample of 17 (8%) women who completed the survey.

**Results:**

The majority of all participants reported being told that participation in the genetic study was voluntary (97%), that they did not feel pressured to participate in the study (99%), and that they could withdraw from the study (81%). The majority of the breast cancer patients (83%) compared to 58% of women in the control group reported that the study purpose was to learn about the genetic inheritance of breast cancer (OR 3.44; 95% CI =1.66, 7.14, p value = 0.001). Most participants reported being told about study procedures (95%) and study benefits (98%). Sixty-eight percent of the patients, compared to 47% of the control group reported being told about study risks (p-value <0.001). Of the 165 married women, 19% reported asking permission from their husbands to enroll in the breast cancer study; no one sought permission from local elders. In-depth interviews highlight the use of persuasion and negotiation between a wife and her husband regarding study participation.

**Conclusions:**

The global expansion of genetic and genomic research highlights our need to understand informed consent practices for studies in ethnically diverse cultural environments such as Africa. Quantitative and qualitative empirical investigations of the informed consent process for genetic and genomic research will further our knowledge of complex issues associated with communication of information, comprehension, decisional authority and voluntary participation. In the future, the development and testing of innovative strategies to promote voluntary participation and comprehension of the goals of genomic research will contribute to our understanding of strategies that enhance the consent process.

## Background

Investigators from multiple disciplines have increased their efforts to use genomic tools to shed light on the complex interplay between genetic and non-genetic factors in disease etiology [1]. The systematic growth of genetic and genomic research initiatives highlights the need to understand and address informed consent practices for genetic studies with diverse populations [2]. Empirical studies on informed consent practices for biomedical research in low or middle-income countries have increased in recent years [3-17] including studies of consent for genetic research in culturally diverse populations in African settings [18-27].

In a series of articles, Tekola, Bull, Farsidesm, Newport Adeyemo, Rotimi, Davey, for example, examined a range of issues associated for genetic research on podoconiosis in Ethiopa, including the potential for social stigma on the process of obtaining consent, and tailoring consent to enhance its effectiveness for study participants [19-21]. The process of informed consent for genetic epidemiological research on hypertension in Nigeria has been studied by Marshall, Adebamowo, Adeyemo and colleagues [22]. Richards and colleagues examined issues surrounding consent in a genetic epidemiological study of women with breast cancer [23]. Marsh and her associates studied community engagement and informed consent in a genetic project on severe childhood diseases in Kenya [24]. Tindana, Bull, Amenga-Etego, de Vries and colleagues have explored the consent process for participation in the MalariaGEN project, a genomic research study, implemented in Ghana [25]. In their discussion of ethical issues raised in human genomics research in developing countries, de Vries, Bull, Doumbo, Ibrahim and colleagues draw on experiences of the MalariaGEN Consortium in developing countries including African sites [26]. Rotimi and Marshall outline specific challenges for informed consent to genetic and genomic research in culturally diverse low-income settings [27].

International and national guidelines for ethical conduct in research address specific requirements for informed consent [28-34]. The process of informed consent centers on voluntary participation and the ability of individuals or their surrogates to comprehend information about study goals and risks. Despite the availability of guidelines, obtaining truly informed consent can be challenging in actual practice [35-40]. Consent documents may be lengthy and difficult to understand, especially when they include sophisticated medical or genetic terms such as haplotype, placebo and randomization [41-44]. Language barriers and requirements for signed consent may diminish effective communication, particularly in areas with low levels of formal education, high illiteracy rates, or where signatures are used infrequently in medical affairs [45,46]. Lack of trust in medical institutions can exacerbate challenges to informed consent and some individuals or communities may also be vulnerable to coercion because of their poverty or social and political conditions that affect voluntary participation [47-49]. Additionally, beliefs about individual autonomy and decision-making capacity are embedded within cultural and social patterns of family ties and community obligations [50-54].

In this paper, we report findings from a study examining factors associated with voluntary participation and comprehension of the study purpose among women involved in genetic epidemiological research on breast cancer in Ibadan, Nigeria, a large metropolitan city. We compare the responses to surveys among women with breast cancer to women in the control group without breast cancer. We anticipated that breast cancer patients might report higher levels of comprehension of the elements of informed consent for the genetic study, compared to women in the control group, because of their more frequent interactions with the health care system. At the time of this study, the population of Ibadan was about 2 million people. Over 90% of Ibadan residents are Yoruba. The area in Ibadan where participants were recruited has an active Council of Elders; regularly scheduled meetings are organized to inform local residents about ongoing activities, including medical research being implemented in the community.

Nigeria is an ethnically diverse country with more than 370 tribes. The three dominant tribes are the Yoruba in the south west, the Igbo in the south east, and the Hausa in the north. The official language of Nigeria is English but more than 500 languages are spoken in Nigeria. It is common for Nigerians to speak local languages in addition to English. The two primary religions in Nigeria are Christianity and Islam; Islam represents the dominant religion of the Hausa in northern Nigeria.

## Methods

We surveyed women who were diagnosed with breast cancer (cases) and women who have never been diagnosed with breast cancer (controls) enrolled in a genetic epidemiological study conducted in Ibadan, Nigeria. This ongoing international collaborative study was designed to identify high risk alleles for breast cancer among Black women on two continents-Africa and North America [23]. Participants signed or placed thumbprints on written consent forms for the genetic study; consent forms were translated into Yoruba and back-translated into English. Consent forms were read to and discussed with all participants by research assistants fluent in English and Yoruba and trained to conduct the process of informed consent.

Patients were recruited at the Oncology Clinics of the University College Hospital, Ibadan, Nigeria, while women in the control group were recruited from a community adjoining the hospital. Participants were enrolled in the informed consent study either soon after recruitment into the genetic epidemiology or at attendance in follow-up clinics. All participants were women 18 years of age or older. Verbal informed consent was obtained from all participants. An Information Sheet explaining the study was given to all participants. The Information Sheet was translated into Yoruba and back-translated into English. Research assistants at the University of Ibadan participated in a training workshop on obtaining informed consent in a culturally and linguistically appropriate manner. Research assistants were fluent in both English and Yoruba. Informed consent was obtained in either Yoruba or English, depending upon individuals’ choice and comfort level with the language.

The research protocol was reviewed and approved by Institutional Review Boards at Loyola University of Chicago, Howard University, Case Western Reserve University, and the University of Ibadan.

### Survey and in-depth interviews

Quantitative and qualitative methodologies were employed in this study. A survey was administered to 215 participants and in-depth audio-taped interviews were conducted with a sub-sample of 17 survey respondents (8%). A table of random numbers was used to select the subgroup invited to participate in the in-depth interview.

The development of both the survey instrument and the in-depth interview guide were based on earlier research on cultural issues surrounding consent to genetic research in Nigeria [54]. The survey and in-depth interview addressed topics associated with the process of consent including: participants’ recall of being told about study goals, procedures, benefits and risks; comprehension of study purpose, voluntary participation, reasons for participating in the study, and permission of husbands or community elders in decisions to join the genetic study. The in-depth interview guides included 15 questions with probes; these questions followed the topics addressed in the survey but were designed to allow further discussion of the survey items.

The survey instrument and in-depth interview guide were translated into Yoruba and back-translated into English. Both instruments were pre-tested in Nigeria to ensure their accuracy in assessing participants’ understanding of issues surrounding informed consent and decision making regarding participation in the genetic epidemiological study of breast cancer. The survey was first pre-tested with research assistants, with each assistant taking turns being the interviewer and the interviewee. The survey was then pre-tested with three women representative of the study population.

The survey and in-depth interviews were conducted in face-to-face-interviews that took place in health clinics. Research assistants conducted the survey and in-depth interviews in Yoruba or English, depending upon the participant’s choice. Participants were compensated for transportation and lost daily wages and provided with a small gift of vitamins. Surveys were administered in approximately thirty minutes. In-depth interviews lasted approximately forty-five minutes to one hour.

The survey interview was conducted after women enrolled in the genetics of breast cancer study. The length of time between our survey and consent for the genetic study ranged from the same day to three weeks. In-depth interviews were conducted approximately two months after individuals’ completed the survey.

### Data analysis

### Survey

Five questions were used to evaluate voluntary participation: 1) participants’ report of whether or not they were told that participation in the genetic study was voluntary; 2) if participants’ felt pressured to participate; and 3) if participants’ understood they could withdraw from the study; 4) if married, did participants ask permission to participate in the genetic study from their husband; and 5) if permission to participate in the genetic study was sought from local community elders. Five measures of comprehension of informed consent are reported. Survey participants were asked if, during the consent discussion, they were told about the study purpose, procedures, benefits, and risks associated with participation. Participants were also asked, “What were you told about the purpose of the study?” Responses to this question were recorded verbatim by trained research assistants conducting the interviews. Discrete codes were developed using traditional methods for text analysis [55-57]. Discrepancies in the application of code categories were resolved in consultation with an investigator not directly involved in the coding. In our analysis, we developed two codes to represent responses to the question on the purpose of the study: 1) to learn about genetic inheritance of breast cancer; and 2) to learn about the prevention and treatment of breast cancer. We then created a dichotomous code (“yes” or “no”) to indicate whether or not participants stated that the purpose of the study was to learn about genetic inheritance of breast cancer. Women whose responses were included in the category “genetic inheritance of disease” made specific reference to genetic inheritance by stating that the study purpose was to “learn how breast cancer is passed down from mother to daughter”, “inherited in the family”, or “to find out if the disease [breast cancer] is in the blood”.

Covariates included respondents’ age, education, marital status, ability to read the consent form, past participation in research, and the time interval between consent to the genetic study and the interview date for this study.

Data reliability was ensured through double entry verification. Survey data were entered into MS Access (Version 11, Microsoft Corporation, Redmond, Washington). Data management and analysis was done using univariate and multivariable techniques (SAS Version 8.1, SAS Institute, Cary, NC). We compared responses using Wilcoxon rank-sum tests for continuous variables and χ^2^ for categorical variables. Age-adjusted logistic regression was used to identify significant variables at p-value of < 0.10. These were then used in multivariate logistic regression models to identify significant predictors of outcomes at a p-value of < 0.05 or those that changed the effect estimate by more than 10%. We report p-values, odds ratios, and 95% confidence intervals.

### In-depth interviews

Audio-tapes from in-depth interviews were transcribed and translated into English when necessary. Identifying names did not appear in the transcriptions. Transcriptions were imported into *Atlas*-*ti*, a computer program for managing text data [58]. Standard procedures for analyzing qualitative data were employed [59,60]. Thematic domains were identified through a process of intense review of transcript data; codes for conceptual categories were developed. Coding discrepancies were resolved in discussion with the investigative team.

## Results

### Survey

Table 1 describes the participants’ demographic characteristics. Women with breast cancer were, on average, eight years older than controls. Most women reported being married (76.7%, 165/215). More than half (56.7%, 122/215) of all participants reported a high school education or higher. Women in the control group were more likely to have more than a high school education compared to breast cancer cases (p-value < .0001). Most participants (78.1%, 168/215) reported being able to read the consent forms and no differences were observed in ability to read the consent form between cases and control subjects. As expected, educational level significantly predicted ability to read the consent form; the multivariate OR comparing those with a high school education or more with those who had less than high school education was 119, 95% CI = 15.0, 939; p-value < 0.001. Only 4% (8/215) of the women reported past participation in medical research. The time interval between consent to the genetic study and our interview was longer for cases (4.5 + 36.1 days) than for controls (0.3 + 3.0 days).

**Table 1:** Demographic characteristics of participants

	**Controls**		**Cases**		**Total**	
	**N** = **109**	**%**	**N** = **106**	**%**	**N** = **215**	
Education*						
< High School	15	13.8	47	44.1	62	28.8
High School	10	9.2	21	19.8	31	14.4
> High School	84	77.1	38	35.9	122	56.7
Age (years)**	37.2 + 9.3		45.3 + 10.8		41.2 + 10.9	
Marital status						
Non-married	27	24.8	23	21.7	50	23.3
Married	82	75.2	83	78.3	165	76.7
Able to read consent form						
Yes	99	90.8	69	65.1	168	78.1
No	10	9.2	39	34.9	47	21.9
Past research participation						
Yes	4	3.7	4	3.8	8	3.7
No	105	96.3	102	96.2	207	96.3
Time interval (days) between consent to genetic study and survey**	0.3 + 3.0		4.5 + 36.1		2.4 + 25.5	

*p-value < .0001.

**Mean/Standard Deviation.

### Voluntary participation

The majority of women (97%, 212/215) reported being told that participation in the genetic study was voluntary and that they did not feel pressured to participate in the genetic study (99%, 210/215). Seventy-nine percent (84/106) of the cases and 83% (90/109) of the controls reported being told they could withdraw from the study at any time; this difference was not statistically significant.

#### Seeking permission from husbands or community elders to participate in the genetic study

Nineteen percent (31/165) of the married women reported they sought permission from their husband before joining the genetic study on breast cancer. In multivariate analysis adjusted for age and educational status, women in the control group were more likely than cases to ask for their husband’s permission (OR = 0.37; 95% CI - 0.15, 0.93, p-value = 0.04); and women with less education were more likely to ask their husband’s permission. None of the respondents reported seeking permission from community elders to participate in the genetics of breast cancer study.

### Comprehension of study purpose

All participants reported being told the purpose of the study during the informed consent discussion. When asked to describe the goal of the study, 70% (151/215) said the purpose was to learn about genetic inheritance of breast cancer and 30% (64/215) said the purpose was to learn about the prevention and treatment of breast cancer. A dichotomous code was created to indicate whether or not participants reported that the study purpose was to learn about genetic inheritance of breast cancer. Table 2 reflects the analysis of the dichotomous coding (“yes” or “no”) indicating whether or not the participant reported that the purpose of the study was to learn about genetic inheritance; in multivariate analysis adjusted for age, education and time interval between the genetic study and consent study, cases were more likely than controls to say that the study was to learn about genetic inheritance of breast cancer (OR 3.44; 95% CI = 1.66, 7.14 p-value = 0.001).

**Table 2:** **Comprehension of study purpose**: “**to learn about genetic inheritance**”

**Purpose: to learn genetic inheritance of breast cancer**	**Controls**		**Cases**		**Total**	
	**N** = **109**	**%**	**N** = **106**	**%**	**N** = **215**	**%**
Yes	63	57.8	88	83.0	151	70.2
No	46	42.2	18	17.0	64	29.8
Age adj. OR	3.83					
95% CI	1.93, 7.61					
MV* OR	3.44					
95% CI	1.66, 7.14					

*Adjusted for age, education, time interval between the original study and consent study.

The majority of the participants (98%, 210/215) reported being told the benefits of the study. The majority of respondents (95%, 205/215) reported they were told about study procedures. In contrast, only 57% (123/215) of the respondents reported being told about the risks of participating in the study; 11% (24/215) said they were not told and 32% (68/215) indicated they could not recall. Significant differences were observed between cases and controls (p-value <0.001); 68% (72/106) of the cases compared to 47% (51/109) of the controls said they were told about risks associated with the study.

### In-depth interviews

In-depth interviews were conducted with 17 participants (10 cases and 7 controls). The cases were, on average, 9 years older than the controls. Educational background was comparable between cases and controls. Overall, 4 women completed primary school, 6 women completed high school, and 7 had college degrees. Fourteen were married. All women were able to read the consent form. None had participated in medical research in the past.

Results of analysis are described for: 1) beliefs about the meaning of voluntary participation; 2) beliefs about the need for seek permission from a husbands or community elders to participate in the study, and 3) comprehension of the purpose of the genetics of breast cancer study. Table 3 illustrates these domains of analysis with quotations from the women interviewed; quotations to illustrate the domains of “voluntary participation” and seeking permission from others” are combined in the table.

**Table 3:** **Thematic domains**: **voluntary participation and seeking permission from others**, **and comprehension of study purpose**

**Voluntary participation and seeking permission from others**	“Voluntary participation is when you decide on your own, not that you want to take permission from somebody before you participate. If you feel like participating and you participate, not that you are being forced to do it.” *Breast Cancer Patient*
	“What I understand by this is that God gave us freedom of choice…so I can make a choice by myself.” Breast Cancer Patient
	“It [joining a study] is a choice nobody is forced [to make] because each participant has a right to withdraw at any point and time.” *Control Group*, *Woman from Community*
	“When you give your consent…you have your own will, from your mind you are interested. But an elder giving consent for you, it is just like the village we have mentioned…you are under compulsion to do it because the *Baale* said we must do it and everybody must do it. It is different from, ‘I want to do it,’ and I participated. The first one is out of free will and the other one is out of compulsion.” *Control Group*, *Woman from Community*
**Comprehension of the purpose of the genetics of breast cancer study**	“[Purpose is to] know whether the breast cancer disease has a genetic origin…to know whether it is a genetic problem.” *Breast Cancer Patient*
	“The researcher told us that it is through [the] blood test that we can know if we have problem with the gene in the body.” *Control Group*, *Woman from Community*
	“Through our genes a child can inherit this disease [breast cancer] from us. You [researcher] will take our blood to know if we have a problem in our body. They want to find out about the gene that relates to my disease.” *Breast Cancer Patient*
	“We all know that the body is made up of cells and each human being has her own peculiar kind of a cell formation and we believe that if a group of people have the same gene…they have similar problems…And this [breast cancer] gene varies from one family to another. So one will be able to know how one can benefit as [an] individual, as family and then as a community.” *Control Group*, *Woman from Community*

### Voluntary participation

Results of analysis indicate two key dimensions of what it means to “participate voluntarily”. First, in their descriptions of the meaning of “voluntary participation”, women called attention to the importance of having the *freedom to choose* whether or not to join the study. Second, most women noted their decision to participate in the study was *not forced or compulsory.*

Thus, discussions with the women about the notion of voluntariness suggest a dialectical relationship between self-determination—on one hand, and the lack of coercion or force—on the other hand. The ability to make one’s own decision must be coupled with the absence of coercion in order for participation to be truly voluntary. Illustrating the overriding importance of free will and individual choice in the absence of coercion, a breast cancer patient said, “To have the free will --you are not compelled to do it; you have your option”. When asked “What does it mean to participate voluntarily, another patient replied “I was not forced to participate. It was from my mind that I decided to participate. [It means] doing something from ones heart [and] not necessarily being told to do so”.

An important aspect of voluntary participation in research is the capacity to withdraw from an ongoing study. From this perspective, the notion of voluntariness in relation to scientific research has a temporal dimension including the possibility of changing one’s mind. Volunteering for a study implies a dynamic engagement with the researchers, an engagement that may involve a decision to withdraw after initially agreeing to participate in a project. Several women mentioned this aspect of voluntariness. For example, when asked what she understood by voluntary participation, a woman in the control group noted, “I am not forced to take part in this study and as far as I am concerned I can, at any stage exclude myself [from] any further involvement”.

#### Seeking permission from one’s husband to participate in the genetic study

Results of analysis of in-depth interviews revealed three dominant themes concerning the husband’s role in participants’ decision to join the genetics of breast cancer research: first, the significance of individual autonomy; second, recognition of the cultural value placed on respect for the authority and status of the husband; and third, the importance of discussion and negotiation in the decision-making process.

Twelve of the 17 women interviewed emphasized their ability to make decisions for themselves, as indicated in the exchange below:

*Patient*: When we were told [about] this research, I told my husband and he was happy with it.

*Interviewer*: What if your husband disapproves of the study or anybody discourages you not to participate, will you still participate?.

*Patient*: The opinion of the husband shouldn’t disturb that of the wife because [she] has her own life to live.

*Interviewer*: What is your husband’s opinion for you to participate in this study?.

*Patient*: He did not force me to participate or not to participate. He said I should go ahead and do it.

Indicative of the strength of personal autonomy and individual free will in relation to participation in the genetic research, five of the women said that even if their husband disapproved of their participation, they would go ahead and join the study. Although individual freedom of choice was the dominant theme expressed by the women interviewed, five of the 17 women said they would not join the study if their husband disapproved.

Interviews highlighted the importance of discussing and negotiating the process of decision making with husbands. Specifically, the interviews call attention to the use of persuasion to influence their husbands’ or others’ opinions about the study and their participation in it. When asked, “What if…your husband disapproves it, will you still participate”, a woman said, “I will appeal to him and I know he will allow me to participate”. In response to the question about whose opinion is most important to consider when deciding to join a study, another woman replied, “My own opinion”. And when asked what she would do if her husband disapproved, she replied, “I will convince [him] and I will go ahead and do it”. Similarly, when asked if she would be willing to participate if her husband or other family members disapproved of the study, a participant indicated she would, “[T] ry to convince them if I know the study is going to benefit me. Maybe they did not see it the way I saw it”.

The following exchange illustrates a participant’s views about normative expectations concerning a husband’s role in Nigerian culture and how this is articulated in the context of decision making regarding study participation:

*Patient*: According to Nigerian culture before you do anything you have to gain the consent of your husband. I just had to inform him and he really helped me.

*Interviewer*: What was his opinion?.

*Patient*: He said, pertaining to myself, that the health talk concerning breast cancer as a woman is okay for me. That if I wanted to go I could go there, no problem was attached to it.

*Interviewer*: Let me ask you, assuming your husband disapproved of it?

*Patient*: Actually, I had made up my mind to go; I just wanted to gain his consent.

### Approval of community elders

When asked about their views concerning the role of elders in approving study participation, the women interviewed reported that there might be rural and urban differences regarding the importance of community elders in the implementation of research. One of the women, for example, made the following observation:

The thing about [a rural] community is to take permission from the elders. Even if the villagers agree [to participate], there must be a word from the elders…maybe because of the way they live, you know, they believe there must be a word from their elders if there is going to be a stranger coming to the village. Then the *Baale* or the Chief would [say] ‘Okay, you can go ahead,’ …I don’t think it is possible to go into a village and carry out a study without the approval of the community leader.

When asked if she thought that people would be able to participate in a study if the elders disapproved, this respondent replied, “Assuming I am living in the village, I will have the full mentality of the village…if the *Baale* says no, there is nothing we can do because we see *Baale* as the small god, you know, when he says ‘No,’ I will have that mentality in me that nobody can do it [participate] because anybody that participates would be like an outcast to the entire village”.

### Comprehension of study purpose

Results of analysis indicated that the women interviewed understood the study purpose was to learn about the role of genetics in breast cancer. A woman in the control group said, “They [the researchers] said they want to see [if] maybe cancer is an inherited disease or if something else [is] causing the disease”. In their descriptions of the study purpose, several women included both genetic inheritance of breast cancer and the prevention of breast cancer. For example, a woman with breast cancer noted that, “The purpose is to enlighten us more about breast cancer and [the] ways by which we can prevent it and how to correct it if there is any. They are trying to find out if the gene in our body has cancer”. Another woman called attention to an underlying long term goal of the study which would contribute to the prevention of breast cancer, “The purpose is that if one had already passed through the [blood] test you asked us to partake in, it is possible for you to know…if it [the disease] is a hereditary one, so that it will not continue to spread”. Combining both genetic inheritance and prevention in their descriptions of the goal of the breast cancer study could reflect the conversational nature of the interview. It may also be a function of the participants’ concern about the prevention of breast cancer generally. It could also reflect the way in which the consent discussion for the genetic study was presented to the women. One rationale for the genetic epidemiology of breast cancer research is to prevent other women from getting the disease in future. Indeed, the consent form stated that participating in this research will help doctors understand the causes of breast cancer and may help prevent the disease in other women.

The women interviewed highlighted concerns about their children and the importance of participating in the study because of how genetic inheritance—in this case, for breast cancer—might influence the health of their children. A breast cancer patient said, “I was told that the researcher would be able to learn the cause of cancer in our body and to learn if it was a hereditary disease from our parents so that [we] would not pass it on to our children”. A woman in the control group reported, “The reason for this research…is to find out about [the] gene in our body which can affect our children in the future”. Another woman from the control group said, “I was told that genetic testing helps to know [about] the disease in one’s body and if they detect any disease in one’s body, we can consult a medical practitioner…because it might be a hereditary disease. Also, genetic testing helps…because this disease can be passed to our own children through our genes”.

## Discussion and conclusion

In this study of consent to genetic epidemiological research on breast cancer in the urban center of Ibadan, Nigeria, the majority of all participants (70%) reported that the study purpose was to learn about genetics and breast cancer. However, breast cancer patients (83%) were more likely than women in the control group (58%) to report that the purpose of the study concerned the genetic inheritance of breast cancer. The overall high level of comprehension among all participants about the genetic purpose of the study could reflect the skill of research staff in explaining study goals during the consent discussion for the genetic research. It could also reflect the relatively high level of education among the participants. The higher level of comprehension among the women with breast cancer about the study purpose, compared to women in the control group, could be attributed to their greater interaction with the health system because of their cancer. Women being treated for breast cancer are asked to complete a number of forms during clinical interactions, including consent forms for treatment interventions. This could have increased their attentiveness to the consent process for research participation in the genetic study. In addition, the diagnosis of cancer may sharply focus the mind of the breast cancer cases on every aspect of their interaction with the health care team, including the research staff, to a higher degree than individuals who are enrolled as controls in the community. This higher level of attention by breast cancer cases may be associated with greater attention to detail and improved comprehension. The breast cancer patients may also ask for more clarifications and ask more questions but we did not examine these issues in this study.

In contrast to findings in this study, results from analysis of data in our study of consent to genetic epidemiological studies of hypertension conducted in metropolitan Chicago in the U.S. and in the rural town of Igbo-Ora, Nigeria, show that only about half of respondents at both sites reported the study purpose was to learn more about the genetic inheritance of hypertension [22]. Differences in comprehension of study purpose between participants in the breast cancer study and the hypertension study may reflect the symbolic power attributed to a disease such as cancer, especially among the women with breast cancer in our study. Breast cancer generally evokes greater concern among women everywhere than illnesses perceived as less threatening such as hypertension, and this could impact comprehension of the study purpose during the consent discussion. Hypertension is more common than breast cancer and thus, more people have personal experience with hypertension or perhaps know others with hypertension. Finally, hypertension is a chronic condition that is treatable with medicines or changes in diet or exercise habits while breast cancer requires more invasive interventions such as surgery, chemotherapy or radiation and the outcomes of treatment may not be favorable in the long-term.

Nearly all of the women in this study recalled being told participation was voluntary and that they did not feel pressured to participate. In-depth interviews reveal two important dimensions of participants’ understanding of “voluntariness”. First, women addressed the need for self-determination—in order for participation to be voluntary, a person must be able to make their own decision about joining a study. Second, women emphasized that a decision could not be coerced or forced by someone else. From this perspective, the act of voluntary participation requires both individual choice and the absence of coercion.

The concepts of individual autonomy and self-determination are foundational in the western ethical practice of informed consent. Yet, individuals everywhere may discuss medical issues, including participation in scientific research, with husbands, family members or others who are important to them. Our survey results indicate that only one-fifth of the married participants sought permission from their husbands to join the genetic study. In contrast, findings from our study on consent to the genetics of hypertension research in the town of Igbo-Ora show that nearly half of the married women reported they sought permission from their husbands [22]. In both of these studies, the survey format asked participants to respond “yes” or “no” to the question, “Did you seek permission to participate in the study from your husband?” Because the survey was not designed to evoke explanations about the meaning of seeking permission to join the study, we are cautious about over-interpreting participants’ reports of “seeking permission” from husbands.

Acquiescence to the husband’s authority in decisions about research participation does not represent normative behavior either in the urban city of Ibadan or the rural setting of Igbo-Ora where we conducted our research. Indicative of the lack of a single normative expectation about husbands’ permission for research participation is the variability of the responses at both sites. In other words, not all rural married women reported seeking their husband’s permission and not all urban women reported they did not need to seek their husband’s permission to join the study. Nevertheless, findings from our breast cancer study, compared to the hypertension study, suggest an urban-rural trend. Moreover, if this study has been conducted with Hausa women who were practicing Muslims living in rural northern Nigeria, they may be more likely to seek husbands’ approval to participate in a study because of the possibility for more conservative views about the hierarchy of decisional authority and its implications for the relationship between wives and husbands.

In-depth interviews with participants in the breast cancer study call attention to the nuanced and complex negotiations in the process of decision making between a wife and her husband. We believe that “seeking permission from a husband” to participate in research represents a more dynamic and fluid process of discussing information about a research project, not a simple request to be “allowed” to participate.

Our findings suggest that in the urban setting of Ibadan, Nigeria, decisions concerning participation in genetic epidemiological research on breast cancer did not involve input from community elders. It is important to note that in the community where the study took place there is a well-established council of elders; regularly scheduled public meetings are held with community members to discuss activities, events, and issues of concern. Our survey asked participants to reply “yes” or “no” to the question, “Did you ask permission of a community elder to join the study?” It could be that women did not seek permission from local elders for practical reasons associated with study recruitment. Women may also have reported not seeking permission for study participation from a community elder because they did not believe that the study represented an investigation that might call for consultation with elders.

Although the approval of elders for research participation was not a concern for participants in our study, international guidelines for biomedical research recognize that, in some areas, investigators may need to consult with community leaders or elders before implementing research projects [30,32]. The views of community leaders may be communicated to local populations through accepted social venues such as council meetings or public events; it is in this public arena where their opinions are likely to influence someone’s decision to join a study.

Findings from our study suggest that participants in the genetic epidemiological study of breast cancer in Ibadan reported a high level of comprehension of study goals and voluntary participation. However, these findings need to be treated cautiously; as Hollowell and her colleagues and others have demonstrated, even highly educated individuals may have difficulty understanding information communicated during the process of consent, and specifically, the differences between research and clinical practice [61-63]. This has particular relevance for the breast cancer patients who participated in our study.

There are several limitations of this study. First, findings may not be generalizable to other African sites. The cultural and social context of the site where genetic research is conducted will vary depending upon local traditions, geographic location, and the goals and procedures of the genetic study itself. For example, our study focused on informed consent to genetic epidemiological research on breast cancer in the urban center of Ibadan, Nigeria. By comparison, a study on informed consent for biobanking of DNA samples conducted in a Xhosa community in rural South Africa may result in different findings. Second, the translation of the instruments from English to Yoruba for Nigeria represents another possible limitation. However, a process of back-translation was implemented and all field staff were fluent in English and Yoruba and trained to conduct the survey and in-depth interviews. Third, in our descriptive study, we did not attempt to assess participants’ knowledge of the difference between research and clinical care. Thus, we do not know the extent to which the patients with breast cancer who participated in the study differentiated between consent for clinical care or research.

Our study contributes to the literature on voluntary participation and comprehension of informed consent to genetic research conducted in African settings. The importance of both quantitative and qualitative empirical research on applications of requirements for informed consent to genetic studies will increase as genetic and genomic investigations continue to expand in Africa [64,65]. Currently, for example, genomic research on a range of topics is being implemented through the H3Africa (Human Heredity and Health in Africa) initiative; the goal of H3Africa, supported with funds from the Wellcome Trust and the National Institutes of Health, is to build capacity for genome science on the continent of Africa [65].

The fundamental goal of consent to research-informed and voluntary participation—applies to all medical and behavioral studies, including genetic investigations. Nevertheless, the nature and purpose of particular scientific studies and their associated risks and benefits vary in complexity and this can influence the process of communicating information during the consent conversation. In some genetic and genomic research, for example, the creation of cell lines, ownership of donated DNA samples and data sharing are key features in the design and implementation of the study. The role of the individual in research, whether as someone with disease who has been recruited as a case participant, or someone proven not to have the disease of interest and therefore enrolled as a control, may also affect engagement with the processes of research in a way that may affect comprehension of consent. A breast cancer patient pays more attention to information being provided about breast cancer than a control does. The level of investment in the information is markedly different.

Future empirical investigations on the process of informed consent will further our knowledge of comprehension and voluntary participation in the context of genetic and genomic research involving complicated issues that have implications for individuals and their communities.

## Competing interests

The authors declare that they have no competing interests.

## Authors’ contributions

PM, CR, CA, and AA contributed to the study design, data analysis and interpretation, and manuscript preparation. TO was responsible for data collection and assisted in manuscript development. TS assisted in the qualitative analysis of the in-depth interview transcripts. JZ conducted data analysis of the survey. All authors read and approved the final manuscript.

## Pre-publication history

The pre-publication history for this paper can be accessed here:

http://www.biomedcentral.com/1472-6939/15/38/prepub
